# The Association between VDR Gene Polymorphisms and Diabetic Retinopathy Susceptibility: A Systematic Review and Meta-Analysis

**DOI:** 10.1155/2016/5305282

**Published:** 2016-11-06

**Authors:** Yun Zhang, Wei Xia, Ping Lu, Huijuan Yuan

**Affiliations:** Department of Endocrinology, Henan Provincial People's Hospital and Zhengzhou University People's Hospital, Zhengzhou, Henan, China

## Abstract

*Aims*. Studies on the associations of vitamin D receptor (VDR) gene polymorphisms with diabetic retinopathy (DR) susceptibility reported conflicting results. A systematic meta-analysis was undertaken to clarify this topic.* Methods*. A systematic search of electronic databases (PubMed, EMBASE, and CNKI) was carried out until March 31, 2016. The pooled odds ratio (OR) and 95% confidence interval (CI) were used to assess the strength of the association.* Results*. A total of 7 studies fulfilling the inclusion criteria were included in this meta-analysis (649 cases and 707 controls). Pooled ORs showed a significant association between FokI polymorphism and DR risk in all the four genetic models (OR = 1.612 (1.354~1.921), 1.988 (1.481~2.668), 1.889 (1.424~2.505), and 2.674 (1.493~4.790) in allelic, dominant, recessive, and additive models, resp., *P*_*Z*_ < 0.01), but not for TaqI or BsmI polymorphism (*P*_*Z*_ > 0.05). Similar results were found in the subgroup analysis. Sensitivity analysis indicated that the results were relatively stable and reliable. Results of Begg's and Egger's tests suggested a lack of publication bias.* Conclusions*. Our meta-analysis demonstrated that DR was significantly associated with VDR gene FokI polymorphism. However, due to the relatively small sample size in this meta-analysis, further studies with a larger sample size should be done to confirm the findings.

## 1. Introduction

Diabetic retinopathy (DR) is regarded as the leading cause of legal blindness in adults, characterized by increased vascular permeability, tissue ischemia, and neoangiogenesis [1, 2]. As one of the most prominent pathological microvascular complications in diabetes, the prevalence of DR in diabetes patients has been estimated at 34.6% and that of proliferative diabetic retinopathy (PDR) has been estimated at 7.0% [3], but the frequency varies in different ethnicities.

It has been established that good diabetes control helps to prevent DR; however, the mechanisms underlying the role of hyperglycemia in DR remain unclear. A subanalysis of the Diabetes Control and Complications Trial (DCCT) showed strong retinopathy transmission in families of patients with severe DR but not in those with nonsevere DR [4], supporting potential involvement of genetic factors in DR. Therefore, it is important to identify the genetic susceptibility factors for DR, which would be helpful to clarify the pathogenesis of DR.

As a secosteroid hormone, vitamin D is acquired and synthesized from the diet and ultraviolet radiation. In addition to its well-known function of maintaining normal homeostasis of calcium and phosphorus, it also has potent nonclassical properties, such as anti-inflammatory, antioxidant, antiangiogenic, and antiproliferative properties [5, 6]. It has been reported that vitamin D could inhibit vascular smooth muscle cell growth in vitro through its antiproliferative action. And vitamin D deficiency has been associated with increased prevalence of retinopathy in young T1DM [7] and T2DM [8] patients. The active form of vitamin D acts through a specific vitamin D receptor (VDR), which is widely expressed in human tissues and organs, including the retina [9]. Therefore, the gene encoding VDR is regarded as a candidate gene involved in DR and has been studied in several populations.

The human VDR gene is located on chromosome 12q13.1, with at least 5 promoter regions and 8 protein-coding exons. Several polymorphisms in the VDR gene have been suggested to be involved in the development of DR. However, the results are conflicting and inconclusive. This may be attributed to the limited sample size and inadequate statistical power, which might affect their reliability. A meta-analysis is a statistical procedure of pooling the data from individual studies, increasing effective sample size, enhancing statistical power of the analysis, and producing a single estimate of an effect [10]. Therefore, we performed a comprehensive meta-analysis to further evaluate the association of VDR gene common polymorphisms with DR susceptibility; we focused on the polymorphisms of FokI (rs10735810), BsmI (rs1544410), ApaI (rs7975232), and TaqI (rs731236), as they had been shown to be highly polymorphic and the most studied polymorphisms.

## 2. Methods

### 2.1. Literature Search

Eligible studies were systematically searched in PubMed, EMBASE, and China National Knowledge Infrastructure (CNKI) databases up to March 31, 2016, with keywords including “diabetes OR diabetic retinopathy” and “VDR OR vitamin D receptor” and “polymorphism OR mutation OR variation OR SNP”. No language restrictions were applied. Additional studies were identified by a hand search for references of original studies and review articles about the association of VDR gene polymorphisms with DR. For detailed search strategies, please see S1 (in Supplementary Material available online at http://dx.doi.org/10.1155/2016/5305282).

### 2.2. Inclusion and Exclusion Criteria

Studies were chosen if they met the following criteria: (1) published studies; (2) evaluated association between VDR polymorphisms and DR risk; (3) a case-control or cohort study based on unrelated individuals; (4) sufficient data for examining odds ratios (ORs) with 95% confidence intervals (CIs); (5) genotype distributions of polymorphism of the control population consistent with Hardy-Weinberg equilibrium (HWE). The most recent article would be used to extract data if the authors published more than one article with the same study data. Case reports, editorials, reviews, abstracts from conferences, republished or duplicate studies, and studies with insufficient information for data extraction were excluded.

### 2.3. Data Extraction and Quality Assessment

Two investigators (Y. Zhang and W. Xia) independently extracted data and both of their results were submitted to a third investigator (P. Lu) for verification. If there were inconsistencies, the three investigators discussed the disagreements to resolve the differences. The following information was collected: (1) the first author's name and publication year; (2) country of origin and ethnicity of samples; (3) sample size and gender ratio (male, %), duration of diabetes and glycosylated hemoglobin (HbA1c) level; (4) genotyping methods and genotype distribution.

The Cochrane recommended case-control study quality assessment tool and the Newcastle–Ottawa Scale (NOS) tools were used to evaluate the quality of the eligible studies.

### 2.4. Statistical Analysis

STATA software 12.0 (STATA Corp., College Station, TX, USA) was used for all statistical analyses. Genotype frequency was assessed by chi-square test in the control group for HWE. The strength of the association between VDR polymorphisms and DR susceptibility was assessed through calculating the pooled odds ratios (ORs) and 95% confidence intervals (CIs) of *Z* test. Four genetics models were used for analyses: allelic model, dominant model, recessive model, and additive model; and the *P* values were corrected for multiple testing using the false discovery rate [11]. *Q* tests and *I* ^2^ statistic were used to test the heterogeneity among studies, and *P*_*Q*_ > 0.10 and *I* ^2^ <  50% were considered to be of low heterogeneity. Sensitivity analysis was conducted by sequentially excluding each study to assess the stability of the results. Publication bias was assessed by Begg's and Egger's tests. *P* < 0.05 was considered significant for all tests.

## 3. Results

### 3.1. Characteristics of Published Studies

**Figure 1:**
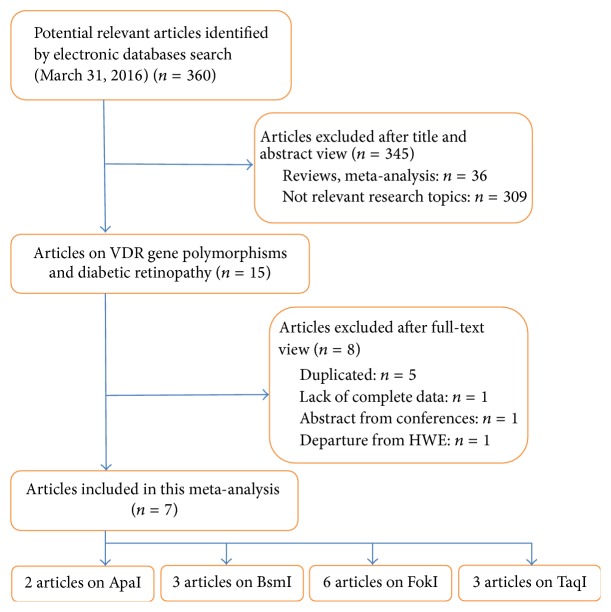
Results of the literature search strategy.

A total of 360 studies were retrieved. Based on titles and/or abstracts, we excluded 36 reviews (or meta-analysis, editorials) and 309 irrelevant studies. As the result in EMBASE was the same as that in PubMed, therefore, 5 studies were retrieved. The remaining 10 studies were included for full-text view. One abstract from conference was excluded. One article was excluded owing to lack of complete data (we tried to contact the author by email and had no response until we submitted our study) [12]. One article was excluded for departure from HWE in the control group [13]. Finally, 7 eligible studies (649 cases and 707 controls) published from 2002 to 2015 were included in this meta-analysis [14–20], and the data was extracted. The study selection procedure was shown in Figure 1. Generally, the major design characteristics of all eligible studies were in accordance with the NOS tool and therefore were of relatively high quality. Reported articles about GWAS of DR were also searched.

Among the 7 studies, 6 studies focused on the association of DR risk and FokI polymorphism [14–19], 3 on TaqI polymorphism [17, 18, 20], 3 on BsmI polymorphism [14, 17, 18], and 2 on ApaI polymorphism [14, 18]. All the polymorphisms were genotyped by polymerase chain reaction-restriction fragment length polymorphism (PCR-RFLP). A total of 4 studies included Caucasian populations [17–20], and 3 included Asian populations [14–16]. Four studies were conducted in type 2 diabetes patients [14–16, 18] and 3 in type 1 diabetes patients [17, 19, 20]. The study characteristics were displayed in Table 1, and the genotype distributions of all studies are summarized in Table 2. The distributions of the genotypes in the control populations were consistent with HWE in all of the studies (*P* > 0.05).

### 3.2. Association of VDR Gene FokI Polymorphism and Risk of DR

Six relevant studies with a total number of 548 cases and 608 controls were included in FokI polymorphism analysis [14–19], 4 in type 2 diabetes patients and 2 in type 1 diabetes patients. The summary results of meta-analysis were shown in Table 3. Pooled ORs showed a significant association between FokI polymorphism and DR risk in all the four genetic models (allelic, dominant, recessive, and additive models). No significant heterogeneity was found except for additive model (Table 3).

**Figure 2:**
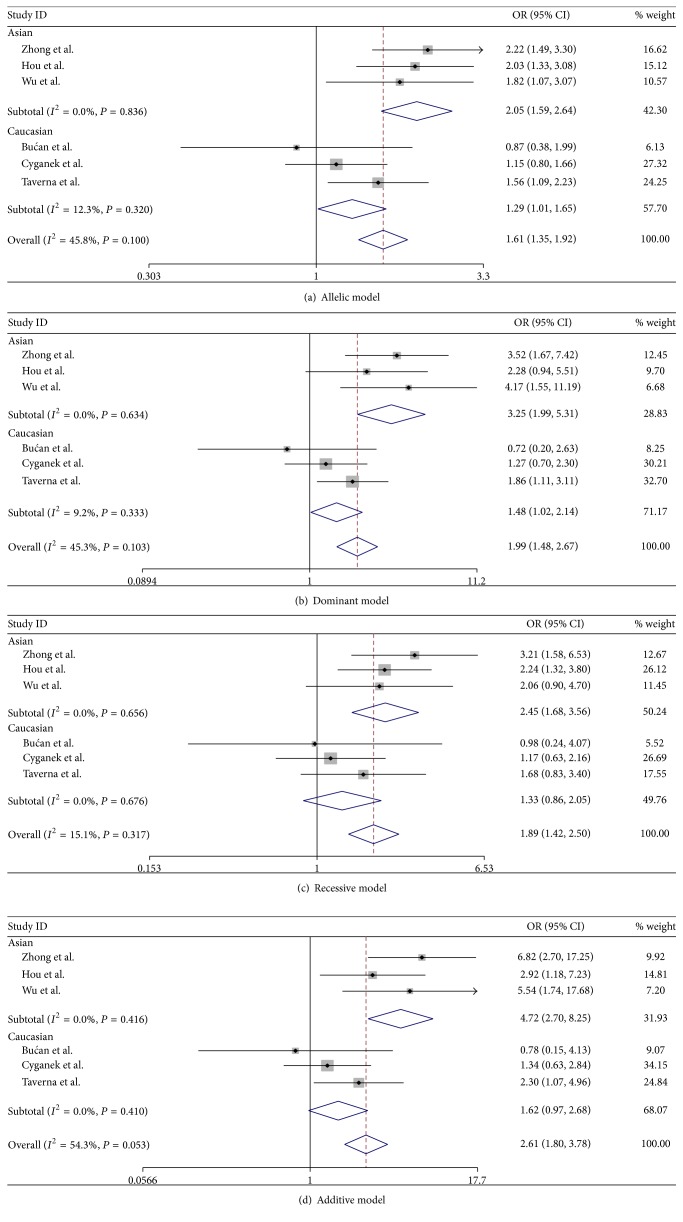
Forest plots for meta-analysis of VDR gene FokI polymorphism and DR risk.

Then, we conducted subgroup analysis stratified by population (Caucasian versus Asian). Overall, heterogeneity in all the four genetic models was not statistically significant either in Asian or in Caucasian populations, and the ORs and 95% CIs were therefore calculated in fixed-effect model. The results indicated that FokI polymorphism was significantly associated with an increased DR risk in all four genetic models (allelic, dominant, recessive, and additive models) in Asian populations; and no significant association was found in Caucasian populations in all the four genetic models (Table 3, Figure 2).

We also conducted subgroup analysis stratified by type of diabetes. A significant association between FokI polymorphism and DR risk in all four genetic models (allelic, dominant, recessive, and additive models) was found in type 2 diabetes patients; and no significant association was found in type 1 diabetes patients in all the four genetic models (Table 3).

### 3.3. Association of VDR Gene TaqI Polymorphism and Risk of DR

Three relevant studies with a total number of 205 cases and 309 controls were included in TaqI polymorphism analysis [17, 18, 20]. Taverna et al. first reported an association between TT genotype and low risk for severe DR in French type 1 diabetes patients [20]. However, no association was found in either study in type 1 diabetes patients by Bućan et al. [17] or study in type 2 diabetes patients by Cyganek et al. [18]. In our meta-analysis, pooled ORs and 95% CIs in four genetic models (allelic, dominant, recessive, and additive models) were 1.145 (0.879~1.492), 1.647 (0.582~4.662), 1.035 (0.600~1.785), and 1.235 (0.689~2.213), respectively. So, no significant association between TaqI polymorphism and risk of DR was suggested.

### 3.4. Association of VDR Gene BsmI Polymorphism and Risk of DR

Three relevant studies with a total number of 198 cases and 320 controls were included in BsmI polymorphism analysis [14, 17, 18]. All the studies were conducted in type 2 diabetes patients. Study by Wu et al. demonstrated a significant association of BsmI genotypes with cumulative prevalence of retinopathy (*P* < 0.05) [16]. However, no association was detected in studies by Zhong et al. [14] or Cyganek et al. [18]. Our meta-analysis showed no significant association between BsmI polymorphism and risk of DR, and heterogeneity was not statistically significant. Pooled ORs and 95% CIs in four genetic models (allelic, dominant, recessive, and additive models) were 1.031 (0.775~1.373), 1.080 (0.579~2.017), 1.025 (0.706~1.487), and 1.130 (0.587~2.175), respectively.

### 3.5. Association of VDR Gene ApaI Polymorphism and Risk of DR

Two relevant studies reported the association between ApaI polymorphism and risk of DR (a total of 179 cases and 292 controls). Study by Zhong et al. demonstrated that VDR gene ApaI polymorphism was not associated with DR risk in Han Chinese type 2 diabetes patients (*P* = 0.92) [14]. The same result was found by Cyganek et al. in Polish type 2 diabetes patients (*P* = 0.23) [18]. Results of meta-analysis showed no significant association between ApaI polymorphism and risk of DR, and heterogeneity was not statistically significant. Pooled ORs and 95% CIs in four genetic models (allelic, dominant, recessive, and additive models) were 1.154 (0.882~1.509), 1.101 (0.710~1.707), 1.372 (0.868~2.169), and 1.308 (0.743~2.303), respectively.

### 3.6. Sensitivity Analysis

In the sensitivity analysis, the influence of each study on the pooled OR was examined by repeating the meta-analysis while omitting each study, one at a time. The results indicated that the overall significance of the ORs was not altered by any single study for all the four genetic models of the FokI polymorphism (Table 4). This indicated that the results of the meta-analysis about VDR gene FokI polymorphism and risk of DR were relatively stable and reliable.

### 3.7. Publication Bias

**Figure 3:**
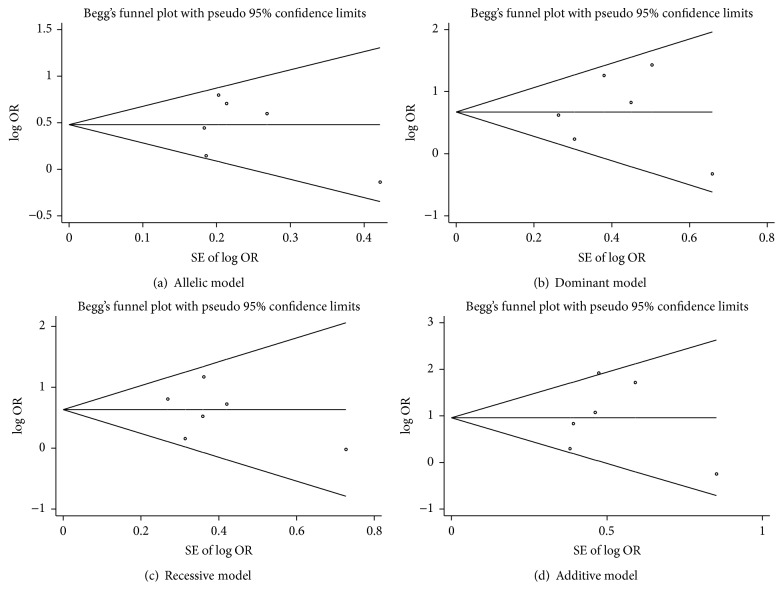
Forest plots for meta-analysis of VDR gene FokI polymorphism and DR risk.

Potential publication bias of the meta-analysis about VDR gene FokI polymorphism and risk of DR was examined by Begg's and Egger's tests. Begg's funnel plot was symmetrical in shape, and the *P* value of Egger's test indicated a lack of publication bias (Table 5 and Figure 3). The results showed no evidence of obvious asymmetry for all the four genetic models.

## 4. Discussion

In the present study, we systematically reviewed all available published studies and performed a meta-analysis to evaluate the association of VDR gene polymorphisms with DR. Seven studies were included in this meta-analysis. Pooled ORs showed a significant association between FokI polymorphism and DR susceptibility in all the four genetic models (allelic, dominant, recessive, and additive models). Sensitivity analysis further showed that the association was stable, and Begg's and Egger's tests indicated a lack of publication bias. Contrary to our meta-analysis, a previous meta-analysis reported no association between FokI polymorphism and DR [21]. We believed our results were more reliable and stable based on four more included studies and larger sample size, thoughtful design, and strict criterion for the included studies.

The most investigated VDR gene polymorphism was FokI polymorphism (rs10735810), a functional T-to-C substitution at exon 2. It abolished the first translation initiation site and resulted in a peptide lacking three amino acids, which influenced the transcriptional activity of VDR [22]. The FF genotype of FokI polymorphism had been associated with higher VDR mRNA copy numbers and increased transcriptional activity of VDR [23]. So, it was presumed that potential beneficial effects of vitamin D on the retina (e.g., immunomodulatory, anti-inflammatory, antioxidant, antiangiogenic, and antiproliferative properties) were higher in patients carrying the F allele than in f-carrier patients. This association had been studied in several populations with conflicting and inconclusive results. So, we preformed this meta-analysis, and our results confirmed the significant association. The results were relatively stable and reliable. In subgroup analysis, the association was limited in Asian populations and in type 2 diabetes patients. The discordancy might be a result of the difference in genetic backgrounds between Asian and Caucasian populations (all the 3 studies were performed in Caucasian populations).

BsmI, ApaI, and TaqI were all located at the 3′ untranslated region of the gene, which was involved in regulation of gene expression, especially through the modulation of mRNA stability [24]. It was interesting to note that significant linkage disequilibrium was found among the TaqI, ApaI, and BsmI polymorphisms [25]. Although these polymorphisms had been reported to be associated with reduced steady state VDR mRNA [26], no association with risk of DR was observed in this meta-analysis. One study in Korean type 2 diabetes patients found that patients with B allele (BB or Bb) of BsmI polymorphism had significant association with lower risk of DR (severe nonproliferative DR or proliferative DR; 7.4%, 5/68) compared with patients without B allele (bb; 17.3%, 81/469; *P* = 0.035) [12]. However, for deficiency of complete data, this study was not included in our meta-analysis.

Some limitations existed in the current meta-analysis that must be considered. First, the conclusion was based on a relatively small number of participants. Therefore, our results might be underpowered. Second, we performed stratification only by race and type of diabetes. The subgroup analysis by type of DR was not performed, because few studies gave data about proliferative DR and nonproliferative DR. Third, our meta-analysis was based on unadjusted estimates without being adjusted for other covariates, such as age, family history, and duration of diabetes. A large, prospective clinical study that includes additional clinical data, such as type of treatment and presence of other microvascular complications, and anthropometric parameters into account is needed to confirm the importance of VDR polymorphism in the development of DR.

## 5. Conclusions

In conclusion, our meta-analysis results indicated that there was a significant association between the VDR gene FokI polymorphism and DR susceptibility. However, due to the relatively small sample size in this meta-analysis, in order to reach a more definitive conclusion, further studies based on larger sample size and substantiation of the variations through functional studies are still needed.

## Supplementary Material

S1 gave the detailed search strategies we used to search in pubmed.

## Figures and Tables

**Table 1:** Characteristics of 7 studies included in this systematic review and meta-analysis.

Author	Publishing year	Population	Ethnicity	Genotyping method	Type of diabetes	Case/control	*n* (case/control)	% of males (case/control)	Mean age (case/control)	Duration of diabetes (case/control)	HbA1c (case/control)
Zhong et al.	2015	Chinese	Asian	PCR-RFLP	DM2	DR/NDR	94/110	46.8/46.4	58.71/57.08	10.0/5.0	9.2/8.35
Hou et al.	2015	Chinese	Asian	PCR-RFLP	DM2	DR/NDR	162/92	48.4/48.9	Not available	Not available	Not available
Wu et al.	2010	Mongolian	Asian	PCR-RFLP	DM2	DR/NC	62/68	51.6/44.1	53.8/51.6	Not available	Not available
Bućan et al.	2009	Croatian	Caucasian	PCR-RFLP	DM1	DR/NDR	19/28	Not available	Not available	Not available	Not available
Cyganek et al.	2006	Polish	Caucasian	PCR-RFLP	DM2	DR/NDR	85/182	42.4/46.7	Not available	14.6/9.7	8.4/7.5
Taverna et al.	2005	French	Caucasian	PCR-RFLP	DM1	SDR/NSDR	126/128	46.8/53.1	40.7/42.9	25.5/25.1	9.02/7.88
Taverna et al.	2002	French	Caucasian	PCR-RFLP	DM1	SDR/NSDR	101/99	44.6/58.6	Not available	30.0/25.2	8.9/8.3

DM1: type 1 diabetes; DM2: type 2 diabetes.

DR: diabetic retinopathy; NDR: diabetes without retinopathy.

SDR: severe diabetic retinopathy (severe nonproliferative DR or proliferative DR); NSDR: nonsevere diabetic retinopathy (no DR or minimal nonproliferative DR).

NC: normal control.

**Table 2:** Genotype frequencies of VDR polymorphisms in 7 studies included in this systematic review and meta-analysis.

Author	Genotype frequencies		Allele frequencies		P-HWE
	Case	Control	Case	Control	
*FokI*	FF/Ff/ff	FF/Ff/ff	F/f	F/f	
Zhong et al.	11/53/30	35/61/14	75/113	131/89	0.113
Hou et al.	10/40/112	12/34/46	60/264	58/126	0.167
Wu et al.	6/37/19	21/35/12	49/75	77/59	0.694
Bućan et al.	6/9/4	7/15/6	21/17	29/27	0.700
Cyganek et al.	20/45/20	51/93/38	85/85	195/169	0.713
Taverna et al.	38/65/23	57/56/15	141/111	170/86	0.826

*BsmI*	Bb/Bb/bb	Bb/Bb/bb	B/b	B/b	
Zhong et al.	5/27/62	6/27/77	37/151	39/181	0.096
Bućan et al.	2/10/7	5/16/7	14/24	26/30	0.431
Cyganek et al.	10/37/38	21/84/77	57/113	126/238	0.791

*TaqI*	TT/Tt/tt	TT/Tt/tt	T/t	T/t	
Bućan et al.	7/8/4	11/12/5	22/16	34/22	0.591
Cyganek et al.	40/38/7	82/81/19	118/52	245/119	0.879
Taverna et al.	27/58/16	42/44/13	112/90	128/70	0.783

*ApaI*	AA/Aa/aa	AA/Aa/aa	A/a	A/a	
Zhong et al.	27/54/13	34/60/16	108/80	128/92	0.205
Cyganek et al.	17/39/29	39/100/43	73/97	278/283	0.178

MAF: minor allele frequency.

HWE: Hardy-Weinberg equilibrium.

**Table 3:** Meta-analysis of VDR gene FokI polymorphism and DR susceptibility.

Genetic model	Pooled method	*P*_*Q*_	*I* ^2^ (%)	OR	95% CI	*P*_*Z*_ (FDR)
*f versus F*						
Overall	Fixed-effect	0.100	45.8	1.612	1.354~1.921	<0.001
Caucasian	Fixed-effect	0.320	12.3	1.293	1.013~1.650	0.056
Asian	Fixed-effect	0.836	0.0	2.049	1.591~2.638	<0.001
DM1	Fixed-effect	0.206	37.6	1.418	1.021~1.969	0.057
DM2	Random-effect	0.079	55.9	1.731	1.261~2.377	0.002

*ff + Ff versus FF*						
Overall	Fixed-effect	0.103	45.3	1.988	1.481~2.668	<0.001
Caucasian	Fixed-effect	0.333	9.2	1.475	1.016~2.142	0.055
Asian	Fixed-effect	0.634	0.0	3.254	1.995~5.308	<0.001
DM1	Fixed-effect	0.183	43.7	1.630	1.012~2.626	0.056
DM2	Random-effect	0.091	53.6	2.396	1.346~4.263	0.005

*ff versus Ff + FF*						
Overall	Fixed-effect	0.317	15.1	1.889	1.424~2.505	<0.001
Caucasian	Fixed-effect	0.676	0.0	1.327	0.857~2.055	0.204
Asian	Fixed-effect	0.656	0.0	2.445	1.680~3.559	<0.001
DM1	Fixed-effect	0.503	0.0	1.514	0.809~2.832	0.205
DM2	Fixed-effect	0.183	38.2	2.001	1.459~2.746	<0.001

*ff versus FF*						
Overall	Random-effect	0.053	54.3	2.674	1.493~4.790	0.002
Caucasian	Fixed-effect	0.410	0.0	1.616	0.974~2.683	0.074
Asian	Fixed-effect	0.416	0.0	4.723	2.702~8.253	<0.001
DM1	Fixed-effect	0.248	25.1	1.893	0.948~3.777	0.078
DM2	Random-effect	0.036	64.8	3.304	1.520~7.185	0.005

**Table 4:** Sensitivity analysis of the meta-analysis on VDR gene FokI polymorphism and DR susceptibility.

Studies	*P*_*Q*_	*I* ^2^ (%)	OR	95% CI	*P*_*Z*_
*f versus F*					
Zhong et al.	0.186	35.3	1.492	1.227~1.814	<0.001
Hou et al.	0.097	49.2	1.539	1.269~1.866	<0.001
Wu et al.	0.061	55.6	1.570	1.173~2.101	0.002^*∗*^
Bućan et al.	0.137	42.6	1.661	1.388~1.987	<0.001
Cyganek et al.	0.291	19.5	1.785	1.461~2.180	<0.001
Taverna et al.	0.057	56.4	1.611	1.171~2.217	0.003^*∗*^

*ff + Ff versus FF*					
Zhong et al.	0.177	36.7	1.770	1.282~2.443	0.001
Hou et al.	0.061	55.7	1.974	1.185~3.287	0.009^*∗*^
Wu et al.	0.155	40.0	1.832	1.344~2.497	<0.001
Bućan et al.	0.151	40.5	2.102	1.552~2.846	<0.001
Cyganek et al.	0.172	37.4	2.301	1.641~3.227	<0.001
Taverna et al.	0.059	56.0	2.066	1.159~3.681	0.014

*ff versus Ff + FF*					
Zhong et al.	0.512	0.0	1.697	1.244~2.313	0.001
Hou et al.	0.258	24.6	1.765	1.263~2.466	0.001
Wu et al.	0.212	31.5	1.866	1.382~2.521	<0.001
Bućan et al.	0.283	20.7	1.942	1.455~2.592	<0.001
Cyganek et al.	0.573	0.0	2.152	1.561~2.968	<0.001
Taverna et al.	0.217	30.7	1.933	1.420~2.630	<0.001

*ff versus FF*					
Zhong et al.	0.201	33.0	2.145	1.425~3.228	<0.001
Hou et al.	0.028	63.2	2.616	1.267~5.401	0.009^*∗*^
Wu et al.	0.058	56.1	2.372	1.263~4.456	0.007^*∗*^
Bućan et al.	0.066	54.7	2.999	1.665~5.401	<0.001^*∗*^
Cyganek et al.	0.141	42.1	3.265	2.122~5.023	<0.001
Taverna et al.	0.029	63.0	2.765	1.306~5.856	0.008^*∗*^

^*∗*^Calculated with random-effect model.

**Table 5:** Publication bias analysis of the meta-analysis on VDR gene FokI polymorphism and DR susceptibility.

Genetic model	Test	*t*	95% CI	*P*
*f versus F*	Begg's test			1.000
	Egger's test	−0.49	−8.290~5.814	0.652

*ff + Ff versus FF*	Begg's test			1.000
	Egger's test	0.21	−5.458~6.371	0.841

*ff versus Ff + FF*	Begg's test			1.000
	Egger's test	−0.55	−6.146~4.101	0.609

*ff versus FF*	Begg's test			0.452
	Egger's test	0.10	−7.641~8.240	0.922
