# Supplementary material for: The Association between VDR Gene Polymorphisms and Diabetic Retinopathy Susceptibility: A Systematic Review and Meta-Analysis
Source: Biomed Res Int. 2016 Nov 6;2016:5305282. doi: 10.1155/2016/5305282 (PMC5116338; doi:10.1155/2016/5305282)
Supplement: Supplementary file 1 — S1 gave the detailed search strategies we used to search in pubmed. [file 5305282.f1.docx]

S1 Detailed search strategies

Unless otherwise stated, search terms are free text terms; MeSH = Medical subject heading (Medline medical index term); exp = exploded MeSH; the dollar sign ($) stands for any character(s); the question mark (?) = to substitute for one or no characters; tw = text word; pt = publication type; sh = MeSH; adj = adjacent.

PUBMED:

1 exp Diabetic Retinopathy/

2 exp Retinal Detachment/

3 exp Retinal Degeneration/

4 exp retinal hemorrhage/ or exp retinal neovascularization/ or exp vitreoretinopathy, proliferative/

5 vitreous detachment/ or vitreous hemorrhage/

6 (eye diseas$ or blindness or visual loss$ or vitrectom$ or cataract$).ti.

7 retina$ detachment$.ti,ab.

8 vitreous haemorrhag$.ti,ab.

9 vitreous hemorrhag$.ti,ab.

10 (macular adj (oedema or edema)).ti,ab.

11 microaneurysm$.ti,ab.

12 neovascular$.ti,ab.

13 fibrous tissue$.ti,ab.

14 (retinopath$ or retinitis or maculopath$).ti.

15 (macula defect$ or macula degeneration$).ti.

16 (macula$ adj (defect$ or degeneration$)).ti,ab.

17 or/2-16

18 exp diabetes mellitus/

19 diabet$.tw.

20 or/18-19

21 17 and 20

22 1 or 21

23 vitamin D receptor OR VDR

24 polymorphism OR mutation OR variation OR SNP OR gene

25 23 and 24

26 FokI OR rs10735810 OR rs2228570

27 BsmI OR rs1544410

28 ApaI OR rs7975232

29 TaqI OR rs731236

30 or/25-29

31 22 and 30
